# Nimotuzumab combined with gemcitabine and nab-paclitaxel as first-line therapy for advanced pancreatic cancer: a single-arm, single-center Phase II prospective study

**DOI:** 10.3389/fmed.2026.1838482

**Published:** 2026-05-29

**Authors:** Lin Lin, Xinyi Zhao, Yawen Luo, Jia-Hua Cai, Zhihuan Lin, Liang Xiao, Geng Tian, Shaozhong He

**Affiliations:** 1 Department of Oncology, The First Affiliated Hospital of Shenzhen University, The Second People’s Hospital of Shenzhen, Shenzhen, China; 2 Shenzhen University, Shenzhen, China; 3 School of Public Health, Southern Medical University, Guangzhou, China; 4 Department of Oncology, Sanya Central Hospital (Hainan Third People’s Hospital), Sanya, China

**Keywords:** advanced pancreatic cancer, first-line therapy, gemcitabine, nab-paclitaxel, nimotuzumab, Phase II, prospective study

## Abstract

**Background:**

Pancreatic cancer remains one of the most lethal malignancies, with a five-year survival rate of merely 8% for exocrine tumors, and conventional chemotherapy offering limited clinical benefit. Nab-paclitaxel plus gemcitabine (AG) is a standard first-line regimen, yet outcomes remain suboptimal. Nimotuzumab, a humanized anti-epidermal growth factor receptor (EGFR) monoclonal antibody with a favorable tolerability profile, has demonstrated promising activity in combination with gemcitabine in KRAS wild-type pancreatic cancer.

**Objective:**

This study aimed to preliminarily evaluate the efficacy and safety of nimotuzumab combined with gemcitabine and nab-paclitaxel (NTZ-AG) as first-line therapy in patients with locally advanced or metastatic pancreatic cancer.

**Methods:**

This single-arm, single-center Phase II prospective study enrolled 16 patients (aged 18–75 years, ECOG PS 0–1) with pathologically confirmed Stage III–IV pancreatic cancer without prior systemic chemotherapy. Patients received nimotuzumab 400 mg (day 1, 3-h infusion), gemcitabine 1,000 mg/m^2^ (days 1 and 8), and nab-paclitaxel 125 mg/m^2^ (days 1 and 8) every 21 days for up to 6 cycles. The primary endpoint was objective response rate (ORR), assessed per RECIST v1.1; secondary endpoints included progression-free survival (PFS), overall survival (OS), clinical benefit rate (CBR), duration of response (DOR), and safety, graded per NCI-CTCAE v5.0.

**Results:**

Among 16 evaluable patients, the ORR was 68.75% (11/16; 95% CI: 42.81–87.34%), with no complete responses and 11 partial responses. The disease control rate (DCR) was 100%. Median PFS was 6.0 months (95% CI: 4.06–7.95) and median OS was 12.0 months (95% CI: 8.35–15.66), with one patient achieving an OS of 49 months. Adverse events were predominantly Grade 1–2; no Grade 4 or higher toxicities were observed. Grade 3 events included alopecia (81.2%), nausea (12.5%), and myelosuppression.

**Conclusion:**

The NTZ-AG regimen demonstrated meaningful anti-tumor activity and an acceptable safety profile as first-line therapy for unselected advanced pancreatic cancer patients, providing a rational basis for larger randomized controlled trials.

**Clinical trial registration:**

This trial was registered at the Chinese Clinical Trial Register (ChiCTR) under the registration number ChiCTR2300072843 having URL https://www.chictr.org.cn/showprojEN.html?proj=198791.

## Highlights

- NTZ-AG (nimotuzumab + nab-paclitaxel + gemcitabine) achieves an ORR of 68.75% and a 100% disease control rate as first-line therapy for advanced pancreatic cancer.
- Median overall survival of 12 months, including one patient achieving 49 months, demonstrates durable anti-tumor activity of the NTZ-AG regimen.
- The addition of nimotuzumab to the AG backbone does not substantially increase toxicity; no Grade ≥4 adverse events or treatment discontinuations were observed.
- NTZ-AG does not require KRAS pre-screening, enabling immediate treatment initiation in settings where molecular diagnostics are unavailable.
- These preliminary data provide a scientific rationale for a multicenter randomized controlled Phase III trial of NTZ-AG versus AG in advanced pancreatic cancer.

## Introduction

Pancreatic cancer is one of the most aggressive malignancies of the gastrointestinal tract, characterized by late-stage presentation, rapid disease progression, and dismal long-term outcomes (1). According to the latest statistics from the American Cancer Society (2026), the five-year relative survival rate for pancreatic cancer is approximately 13% overall, decreasing to a mere 8% when considering only exocrine tumors—primarily pancreatic ductal adenocarcinoma (PDAC)—which account for approximately 92% of all cases (2, 3). These figures underscore the profound clinical challenge that pancreatic cancer continues to pose despite decades of therapeutic advancement.

Radical surgical resection remains the only potentially curative intervention for pancreatic cancer. However, due to the insidious onset of symptoms and the absence of reliable early diagnostic biomarkers, only 15–20% of patients are eligible for surgery at the time of diagnosis (4). The majority present with locally advanced or metastatic disease: approximately 30–35% have locally advanced pancreatic cancer (LAPC), and approximately 50% have distant metastases at initial evaluation, placing them beyond the reach of curative resection (5, 6).

For patients with unresectable or metastatic disease, systemic chemotherapy constitutes the cornerstone of treatment. Since its approval by the U.S. Food and Drug Administration (FDA) in 1997, gemcitabine has served as the standard first-line agent for advanced pancreatic cancer (7). However, gemcitabine monotherapy offers only modest clinical benefit, with a median overall survival of approximately 5–6 months and an objective response rate below 10% (8). The landmark MPACT Phase III trial demonstrated that the combination of nab-paclitaxel and gemcitabine (the AG regimen) significantly improved survival compared to gemcitabine monotherapy, with a median OS of 8.5 months versus 6.7 months, thereby establishing AG as one of the standard first-line regimens (9). Nevertheless, meaningful progress remains elusive, and the development of novel therapeutic strategies with superior efficacy and manageable toxicity profiles is of paramount clinical importance.

Molecular-targeted therapy has emerged as a research priority in advanced pancreatic cancer, owing to its potential for selective anti-tumor activity with reduced systemic toxicity. The epidermal growth factor receptor (EGFR) is overexpressed in 30–89% of pancreatic cancers, where its aberrant activation drives tumor cell proliferation, invasion, and metastasis, positioning it as an attractive therapeutic target (10). Erlotinib, a small-molecule EGFR tyrosine kinase inhibitor, in combination with gemcitabine, was the first targeted agent approved for advanced PDAC; however, its survival benefit was marginal and its use is now largely limited (11). Anti-EGFR monoclonal antibodies represent an alternative strategy with the potential for more selective receptor blockade.

Nimotuzumab is a humanized IgG1 monoclonal antibody directed against the extracellular domain of EGFR. Its distinctive intermediate binding affinity confers selective targeting of tumor cells overexpressing EGFR, while minimizing off-target effects on normal tissues—a pharmacological property that translates into a favorable tolerability profile with notably low rates of skin toxicity and infusion-related reactions compared to other anti-EGFR antibodies such as cetuximab (12). Preclinical and clinical data suggest that nimotuzumab inhibits EGFR-mediated downstream signaling cascades, including the RAS/RAF/MAPK and PI3K/AKT/mTOR pathways, thereby suppressing tumor cell proliferation and inducing apoptosis. Furthermore, nimotuzumab may sensitize tumor cells to gemcitabine by downregulating DNA repair mechanisms, offering a mechanistic rationale for combination therapy (13).

Several clinical studies have begun to validate the efficacy and safety of nimotuzumab in combination with chemotherapy for advanced pancreatic cancer. The German PCS07 Phase IIb trial demonstrated that nimotuzumab plus gemcitabine improved OS compared to gemcitabine monotherapy (8.6 vs. 6.0 months; HR = 0.69), with a particularly striking benefit in the KRAS wild-type subgroup (hazard ratio = 0.32, representing a 68% reduction in the risk of death) (14). Building on this observation, the Chinese NOTABLE Phase III trial—specifically designed for KRAS wild-type patients—confirmed that nimotuzumab plus gemcitabine prolonged OS (10.9 vs. 8.5 months; HR = 0.66) and improved PFS, with a well-tolerated safety profile (15). These pivotal findings provided a compelling scientific basis for further investigation of nimotuzumab in combination with more intensive chemotherapy backbones.

In a preliminary exploratory cohort conducted by our research group, nine patients with advanced pancreatic cancer treated with the triple combination of nimotuzumab, nab-paclitaxel, and gemcitabine achieved an objective response rate of 80% and a median OS of 20 months, with acceptable tolerability. Motivated by these encouraging preliminary data, the current study was designed as a formal single-arm, single-center Phase II prospective clinical trial to systematically evaluate the efficacy and safety of the NTZ-AG regimen (nimotuzumab + nab-paclitaxel + gemcitabine) as first-line therapy for locally advanced or metastatic pancreatic cancer, with the aim of generating evidence to support future randomized controlled studies and to expand treatment options for this patient population.

## Patients and methods

### Study design

This was a single-arm, single-center, open-label Phase II prospective clinical study initiated and conducted at the Second People’s Hospital of Shenzhen, China. The study was designed to evaluate the efficacy and safety of nimotuzumab in combination with gemcitabine and nab-paclitaxel as first-line therapy for locally advanced or metastatic pancreatic cancer. The study protocol was developed in accordance with the ethical principles of the Declaration of Helsinki and Good Clinical Practice (GCP) guidelines, and was reviewed and approved by the Clinical Research Ethics Committee of Shenzhen Second People’s Hospital (ethics approval number: 2023-092-02PJ) prior to patient enrollment. All participants provided written informed consent after being fully informed of the study objectives, procedures, potential risks, and available alternatives.

**Figure 1:**
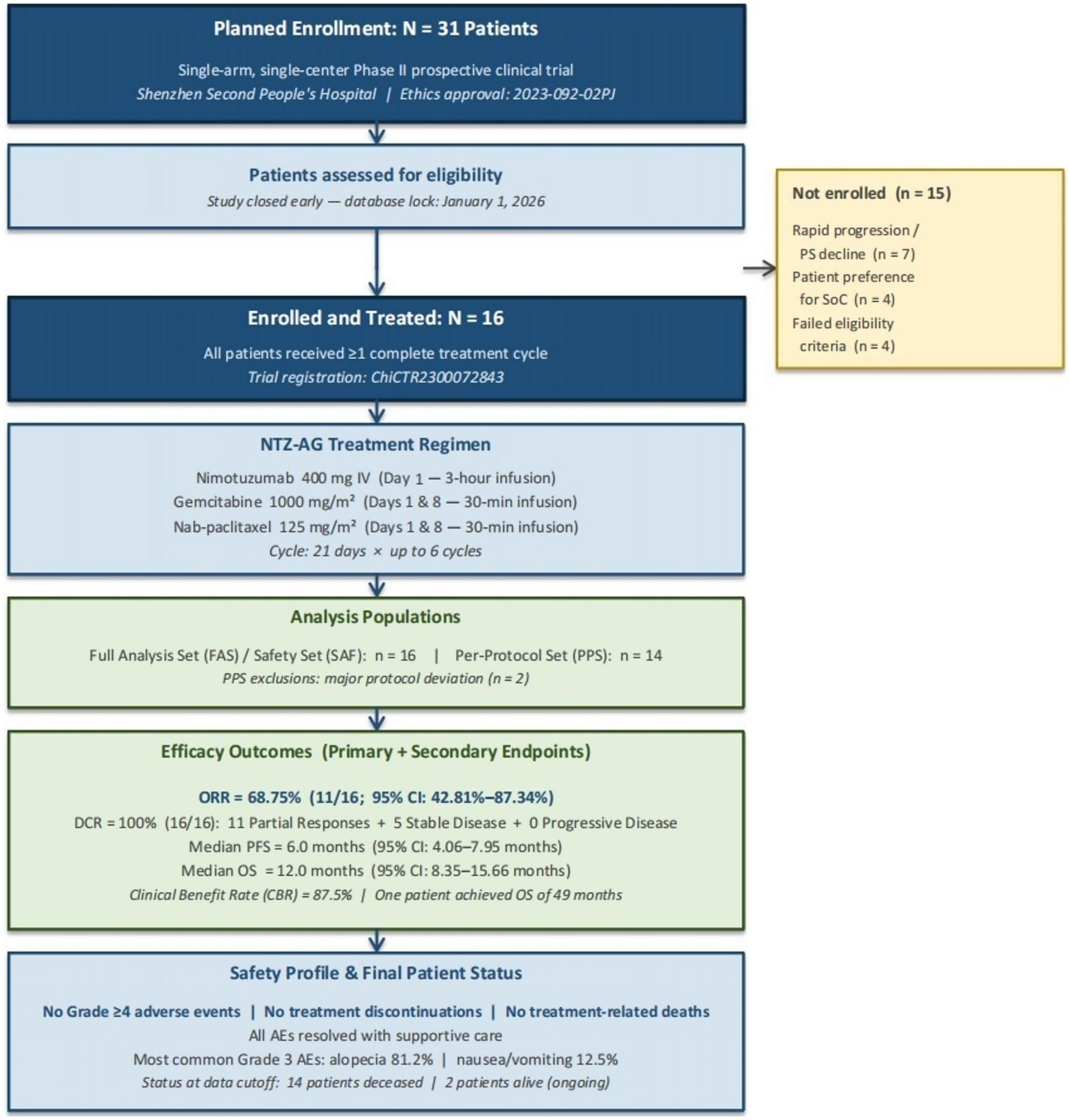
Flow diagram of patient enrollment, treatment, and analysis (*N* = 16). FAS = Full Analysis Set; SAF = Safety Analysis Set; PPS = Per-Protocol Set; NTZ-AG = nimotuzumab + *nab*-paclitaxel + gemcitabine.

Given the single-arm design, randomization was not applicable; all eligible patients were enrolled into a single treatment group (Figure 1). Clinical data were collected and managed according to CDISC standards with rigorous data cleaning procedures. Adverse events were coded using the Medical Dictionary for Regulatory Activities (MedDRA), and concomitant medications were coded using the World Health Organization Drug Dictionary (WHO-DD). Primary endpoint tumor assessments were conducted through independent blinded imaging review by two radiologists not involved in clinical care, who evaluated imaging studies independently according to RECIST v1.1; discordant assessments were adjudicated through consensus discussion. The trial was registered at the Chinese Clinical Trial Register under the registration number ChiCTR2300072843 (Figure 1).

### Patient selection

Eligible patients were required to meet all of the following inclusion criteria: (1) histologically or cytologically confirmed locally advanced (Stage III) or metastatic (Stage IV) pancreatic cancer with no prior systemic chemotherapy; for patients who declined or were not amenable to pancreatic biopsy, diagnosis could be established based on PET-CT, magnetic resonance imaging (MRI), and tumor markers (CA19-9, CA125, CEA) according to the 2022 CSCO Pancreatic Tumor Diagnosis and Treatment Guidelines (16) (2) age 18–75 years; (3) Eastern Cooperative Oncology Group (ECOG) performance status score of 0–1; (4) expected survival of ≥3 months; (5) at least one measurable lesion per RECIST v1.1; and (6) adequate hematologic function (white blood cell count ≥4 × 10^9^/L, absolute neutrophil count ≥1.5 × 10^9^/L, platelet count ≥100 × 10^9^/L, hemoglobin ≥90 g/L), renal function (serum creatinine ≤1.2 mg/dL or creatinine clearance ≥60 mL/min), and hepatic function (total serum bilirubin ≤1.5 × ULN [≤3.0 × ULN for hepatic metastases]; AST and ALT ≤2.5 × ULN [≤5.0 × ULN for hepatic metastases]).

Patients were excluded if they met any of the following criteria: participation in another interventional clinical trial within 30 days before enrollment; concurrent diagnosis of another untreated malignancy; uncontrolled comorbidities (including cardiac failure, poorly controlled diabetes mellitus, hypertension, thyroid disorders, or significant psychiatric illness); known HIV infection or active viral hepatitis; chronic corticosteroid use (prednisone >10 mg/day or equivalent) for more than 6 months; known hypersensitivity to any component of the study regimen; grade ≥2 peripheral neuropathy or hearing loss per NCI-CTCAE v5.0; pregnancy (confirmed by serum or urine HCG) or lactation, or unwillingness to use effective contraception for at least 6 months after the last treatment dose; or any other condition judged by the investigator to preclude safe participation or preclude the ability to provide informed consent.

### Treatment regimen

Patients received the NTZ-AG regimen consisting of: nimotuzumab 400 mg administered as a 3-h intravenous infusion on day 1 of each cycle; gemcitabine 1,000 mg/m^2^ administered as a 30-min intravenous infusion on days 1 and 8; and nab-paclitaxel 125 mg/m^2^ administered as a 30-min intravenous infusion on days 1 and 8. Each treatment cycle was 21 days in duration. Treatment was continued for up to 6 cycles or until disease progression, occurrence of unacceptable toxicity, patient withdrawal of consent, or investigator decision to discontinue therapy, whichever occurred first. For Grade 1–3 adverse events, treatment was continued at the original doses following symptomatic management. In the event of potential Grade 4 or higher adverse events (none of which were actually observed in this study), treatment would be temporarily suspended and doses adjusted or therapy discontinued after adverse events recovered to Grade ≤1.

### Efficacy assessment

Tumor response was assessed according to RECIST v1.1 (17). A baseline tumor imaging assessment (contrast-enhanced CT or MRI of the chest, abdomen, and pelvis, encompassing all known metastatic sites) was performed within 2–3 weeks before treatment initiation; the same equipment and standardized scanning parameters (slice thickness ≤5 mm) were used at each time point to ensure comparability. During the treatment period, imaging assessments were repeated every 2 cycles (approximately every 42 days); after completion of 6 treatment cycles, assessments were scheduled every 3 cycles (approximately every 63 days) until disease progression, treatment discontinuation, or loss to follow-up. Tumor response was categorized as complete response (CR), partial response (PR), stable disease (SD), or progressive disease (PD). For patients with an initial assessment of CR or PR, confirmatory imaging was required at least 4 weeks after the initial assessment to avoid false-positive results due to imaging variability. Patients with clinical deterioration suggestive of disease progression (worsening pain, declining performance status) could undergo early imaging assessment at the investigator’s discretion. Patients who withdrew from the study were required to undergo a final imaging assessment if the last scheduled assessment was more than 4 weeks prior to discontinuation.

### Safety monitoring

Safety monitoring commenced from the time of informed consent and continued throughout the study period. All adverse events (AEs) were recorded, including event name, onset date, severity, management, and outcome. At the beginning of each treatment cycle, prior to nimotuzumab administration, patients underwent assessment of vital signs, ECOG performance status, and thyroid function (FT3, FT4, TSH). On days 1 and 15 of each 21-day cycle, complete blood counts, urinalysis, serum biochemistry (including liver and renal function, electrolytes, and cardiac enzymes), and coagulation parameters were evaluated. Adverse events were graded per NCI-CTCAE v5.0 and summarized by System Organ Class (SOC) (18). The causal relationship between each AE and the study drug was assessed using a five-category classification (definitely related, probably related, possibly related, possibly unrelated, unrelated).

### Study endpoints

The primary endpoint was the objective response rate (ORR), defined as the proportion of patients achieving CR or PR per RECIST v1.1, with confirmation required no earlier than 4 weeks after initial assessment. Secondary endpoints included: progression-free survival (PFS), defined as the time from enrollment to the first documented evidence of tumor progression or death from any cause, whichever occurred first; clinical benefit rate (CBR), defined as the proportion of patients achieving CR, PR, or SD lasting ≥6 months; duration of response (DOR), defined as the time from first documented CR or PR to disease progression or death; overall survival (OS), defined as the time from enrollment to death from any cause; and safety endpoints including the incidence, severity, and resolution of adverse events.

### Statistical analysis

Three analytical datasets were defined. The Full Analysis Set (FAS) included all patients who signed informed consent, received at least one dose of study treatment, and had baseline tumor assessments; the FAS served as the primary efficacy analysis population, following the intention-to-treat (ITT) principle, with patients lacking post-baseline tumor assessment classified as non-responders for ORR analyses. The Per-Protocol Set (PPS) further restricted analysis to FAS patients who met all major eligibility criteria, showed adequate adherence, had no major protocol deviations, and had evaluable primary endpoint data; the PPS was used for supportive and sensitivity analyses. The Safety Analysis Set (SAF) included all patients who received at least one dose of study treatment and had post-treatment safety data, and was used for all safety analyses (19).

Sample size was calculated based on an expected ORR of 23% for the AG regimen alone (9) and a hypothesized improvement to 43% with the addition of nimotuzumab (absolute increase of 20%). Using a one-sample binomial proportion test with a two-sided *α* = 0.05 and *β* = 0.20 (power = 80%), implemented in PASS 15 software, the required sample size was estimated at 31 patients.

For survival data (PFS and OS), patients who were lost to follow-up, withdrew consent, or discontinued for non-progression reasons without experiencing an event were censored at the date of last follow-up or last evaluable imaging assessment. All statistical analyses were performed using IBM SPSS Statistics version 27.0. All tests were two-sided, with a significance level of *α* = 0.05 and 95% bilateral confidence intervals. Continuous variables were described using mean, standard deviation, median, interquartile range, minimum, and maximum; categorical variables were described as counts and percentages. ORR was presented as a proportion with 95% CI calculated by the Clopper-Pearson method. PFS and OS were estimated using the Kaplan–Meier method, with median survival times and corresponding 95% CIs reported. Exploratory analyses of factors potentially influencing survival were conducted using Cox proportional hazards regression; all exploratory analyses were hypothesis-generating only, and no adjustments for multiple comparisons were made. Given the final enrolled sample size of 16 patients (51.6% of the planned 31), post-hoc power was 77.06% to detect the observed ORR of 68.75% versus the historical null of 23% (two-sided *α* = 0.05, Cohen’s h = 0.9548).

## Results

### Patient characteristics and enrollment

The study originally planned to enroll 31 patients. However, due to a combination of factors—including rapid disease progression and functional decline during the screening period in some patients, patient preference for standard-of-care chemotherapy, strict eligibility criteria, and the single-center recruitment limitation—only 16 patients were enrolled by the database lock date of January 1, 2026, necessitating early termination of the study without reaching the pre-specified sample size. No pre-planned interim analysis or sample size re-estimation was performed. During the study period, 31 patients were screened for eligibility. Fifteen patients were not enrolled for the following reasons: rapid disease progression or functional decline during the screening period (*n* = 6), patient preference for standard-of-care AG chemotherapy (*n* = 4; none of these patients cited concerns regarding added toxicity or the 3-h nimotuzumab infusion), ineligibility due to ECOG performance status >1 or inadequate organ function (*n* = 3), and withdrawal of consent (*n* = 2). Despite the reduced sample size, the observed ORR of 68.75% was highly statistically significant against the historical 23% benchmark (exact binomial *p* = 0.00013; post-hoc power 77.06%). All 16 enrolled patients had metastatic (Stage IV) pancreatic cancer and were recruited from the Second People’s Hospital of Shenzhen. All patients received at least one complete cycle of treatment and were included in both the FAS and the SAF; 14 patients met criteria for inclusion in the PPS. Baseline demographic and clinical characteristics are summarized in Table 1.

**Table 1:** Baseline characteristics of patients with metastatic pancreatic cancer (*N* = 16).

Characteristic	Value
Age, years	
Median (range)	62 (45–75)
<65	9 (56.2%)
≥65	7 (43.8%)
Sex	
Male	10 (62.5%)
Female	6 (37.5%)
Stage	
IV	16 (100.0%)
Pancreatic tumor location	
Head	7 (43.8%)
Neck	1 (6.2%)
Body	5 (31.2%)
Tail	3 (18.8%)
Site of metastasis	
Liver	12 (75.0%)
Peritoneum	7 (43.8%)
Abdominal cavity	4 (25.0%)
Lung	1 (6.3%)
Number of metastatic sites	
Single site	10 (62.5%)
Multiple sites (≥2)	6 (37.5%)
AG treatment cycles	
Median (range)	6 (1–6)

AG, nab-paclitaxel plus gemcitabine.

The enrolled patients had a median age of 62 years (range: 45–75 years), with 9 patients (56.2%) aged <65 years and 7 (43.8%) aged ≥65 years. The cohort comprised 10 males (62.5%) and 6 females (37.5%). All patients presented with Stage IV disease. Pancreatic tumor location was distributed across the head (*n* = 7, 43.8%), body (*n* = 5, 31.2%), tail (*n* = 3, 18.8%), and neck (*n* = 1, 6.2%). Hepatic metastases were the most common site of distant spread (*n* = 12, 75.0%), followed by peritoneal metastases (*n* = 7, 43.8%), abdominal cavity involvement (*n* = 4, 25.0%), and pulmonary metastases (*n* = 1, 6.3%). Ten patients (62.5%) had involvement of a single metastatic site, while 6 (37.5%) had multiple metastatic sites (≥2). The median number of AG treatment cycles received was 6 (range: 1–6).

### Primary endpoint: objective response rate

Per RECIST v1.1 assessment in all 16 evaluable patients, no patient achieved a complete response (CR), while 11 patients achieved a partial response (PR), and 5 patients had stable disease (SD). No patient experienced primary disease progression (PD). The ORR (CR + PR) was 68.75% (11/16; 95% CI: 42.81–87.34%), and the disease control rate (DCR = CR + PR + SD) was 100% (16/16). All 11 partial responses were confirmed by follow-up imaging performed at least 4 weeks after the initial assessment, in full accordance with RECIST v1.1 guidelines. Efficacy outcomes are summarized in Table 2.

**Table 2:** Efficacy outcomes in patients receiving NTZ-AG (*N* = 16).

Efficacy variable	NTZ-AG *N* = 16
ORR, *n* (%)	11 (68.75)
CR, *n* (%)	0 (0)
PR, *n* (%)	11 (68.75)
SD, *n* (%)	5 (31.25)
PD, *n* (%)	0 (0)
Not evaluable, *n* (%)	0 (0)
No postbaseline assessment, *n* (%)	0 (0)
DCR, *n* (%)	16 (100)
PFS, median (95% CI), months	6.0 (4.06–7.95)
OS, median (95% CI), months	12.0 (8.35–15.66)
OS rate, %	
3 months	100
6 months	75
9 months	61.4
12 months	40.9

ORR, objective response rate; CR, complete response; PR, partial response; SD, stable disease; PD, progressive disease; DCR, disease control rate; PFS, progression-free survival; OS, overall survival; CI, confidence interval; NTZ-AG, nimotuzumab plus nab-paclitaxel and gemcitabine.

### Secondary endpoints: survival outcomes

Progression-free survival (PFS) was estimated by the Kaplan–Meier method. The median PFS (mPFS) was 6.0 months (95% CI: 4.06–7.95 months), with a mean PFS of 8.625 months (95% CI: 5.50–11.75 months). The distribution of PFS ranged from 3 to 27 months; notably, one patient achieved a PFS of 27 months and two patients maintained progression-free survival for more than 15 months, reflecting durable disease control in a subset of patients.

**Figure 2:**
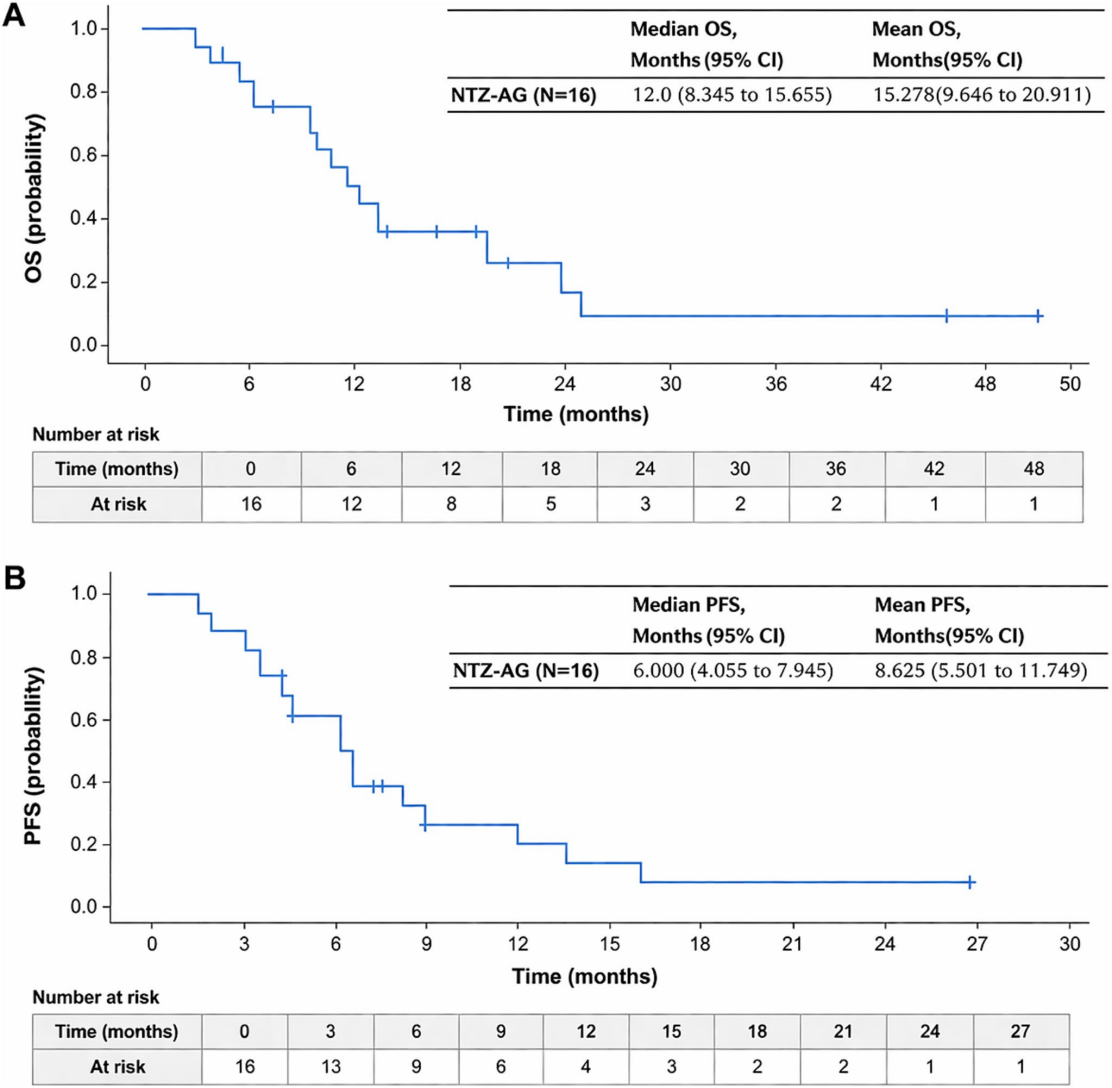
Kaplan–Meier estimates of overall survival **(A)** and progression-free survival **(B)** in patients receiving the NTZ-AG regimen (*N* = 16). Number at risk is shown below the *x*-axis for both OS and PFS curves. The median follow-up time (reverse Kaplan–Meier) was 13.5 months. Only 2 patients remained alive at the data lock date.

As of the data lock date of January 1, 2026, 2 of the 16 patients remained alive. The median OS (mOS) was 12.0 months (95% CI: 8.35–15.66 months), with a mean OS of 15.28 months (95% CI: 9.65–20.91 months). OS ranged from 5 to 49 months; one patient achieved an OS of 49 months and was still alive at the time of analysis, and 4 patients had OS exceeding 20 months. The patient achieving the longest OS (49 + months and still alive at data lock) was a 62-year-old male with an ECOG performance status of 0, a primary tumor located in the pancreatic body, and a single hepatic metastasis. He attained a confirmed partial response after the first two cycles of NTZ-AG and maintained durable disease control throughout treatment. The OS rates at 3, 6, 9, and 12 months were 100, 75, 61.4, and 40.9%, respectively. The median follow-up duration for the entire cohort, estimated by the reverse Kaplan–Meier method, was 13.5 months. Kaplan–Meier survival curves for OS and PFS are presented in Figure 2. Exploratory subgroup analyses by age (<65 vs. ≥ 65 years) and sex showed no clinically meaningful differences in ORR or median OS (Supplemental Table S1).

### Secondary endpoints: clinical benefit rate and duration of response

The clinical benefit rate (CBR), defined as CR, PR, or SD lasting ≥6 months, was 87.5% (14/16). Among the 11 PR patients, the median duration of response (mDOR) had not been reached at the time of data cutoff. Eight PR patients maintained their response for more than 6 months and 3 PR patients sustained their response for more than 12 months, indicating that the anti-tumor activity conferred by the NTZ-AG regimen was not only robust but also durable in a meaningful proportion of patients.

### Safety profile

**Figure 3:**
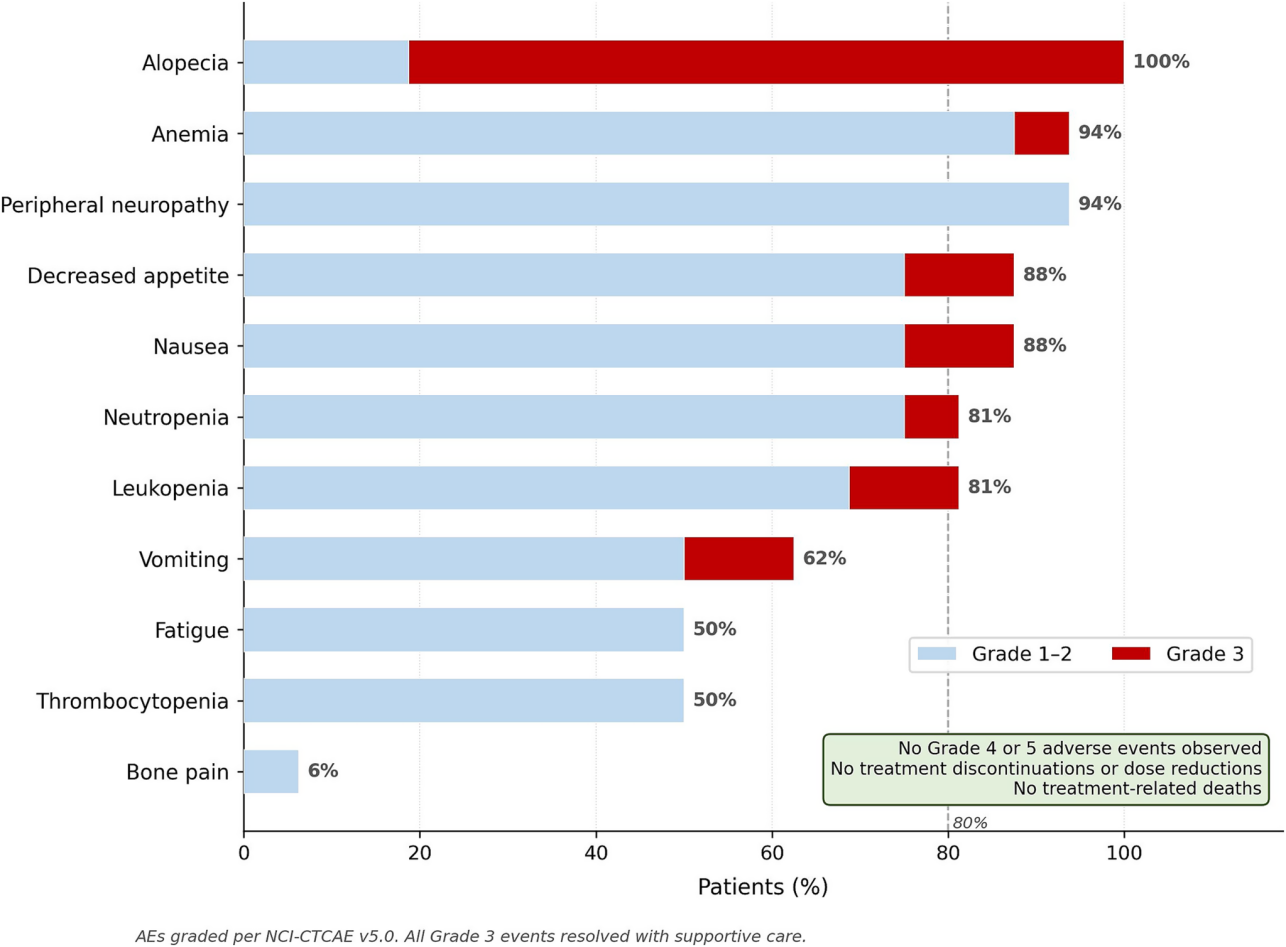
Treatment-emergent adverse events by grade in patients receiving the NTZ-AG regimen (*N* = 16). Bars represent the proportion of patients experiencing each event, stratified by Grade 1–2 (light blue) and Grade 3 (red). No Grade ≥4 adverse events, treatment discontinuations, or treatment-related deaths were observed. AEs graded per NCI-CTCAE v5.0.

All 16 patients (100%) underwent complete safety evaluation throughout the treatment period. All patients experienced at least one treatment-emergent adverse event (TEAE), predominantly of Grade 1–2 severity. No Grade 4 or higher adverse events were observed, and no treatment-related deaths occurred. All adverse events resolved or returned to baseline following appropriate supportive care, including granulocyte colony-stimulating factor (G-CSF) for myelosuppression, 5-HT_3_ receptor antagonists for nausea and vomiting, and nutritional support as needed. No treatment interruptions or permanent discontinuations due to adverse events were recorded, confirming the acceptable tolerability of the NTZ-AG regimen. The overall incidence of adverse events is summarized in Table 3 and Figure 3.

**Table 3:** Overall incidence of adverse events in this trial (*N* = 16).

System	Adverse event	Grade 1, *n* (%)	Grade 2, *n* (%)	Grade 3, *n* (%)	Total, *n* (%)
Gastrointestinal toxicity	Nausea	10 (62.5)	2 (12.5)	2 (12.5)	14 (87.5)
	Decreased appetite	11 (68.8)	1 (6.2)	2 (12.5)	14 (87.5)
	Vomiting	7 (43.8)	1 (6.2)	2 (12.5)	10 (62.5)
	Diarrhea	—	—	—	—
	Constipation	—	—	—	—
Hematological toxicity	Anemia	12 (75.0)	2 (12.5)	1 (6.2)	15 (93.8)
	Leukopenia	8 (50.0)	3 (18.8)	2 (12.5)	13 (81.2)
	Neutropenia	10 (62.5)	2 (12.5)	1 (6.2)	13 (81.2)
	Thrombocytopenia	6 (37.5)	2 (12.5)	—	8 (50.0)
Dermatological toxicity	Alopecia	—	13 (81.2%)	0 (0%)	13 (81.2)
Neurological toxicity	Peripheral neuropathy	11 (68.8)	4 (25.0)	—	15 (93.8)
General symptoms	Fatigue	7 (43.8)	1 (6.2)	—	8 (50.0)
Hepatic dysfunction	Increased ALT	—	—	—	—
	Increased AST	—	—	—	—
Others	Bone pain	1 (6.2)	—	—	1 (6.2)

AE, adverse event; ALT, alanine aminotransferase; AST, aspartate aminotransferase. Grading per NCI-CTCAE v5.0. (—) indicates no events observed at that grade.

High-frequency adverse events (incidence ≥80%) included gastrointestinal toxicities (nausea, 87.5%; decreased appetite, 87.5%), hematological toxicities (anemia, 93.8%; leukopenia, 81.2%; neutropenia, 81.2%), dermatological toxicity (alopecia, 81.2%), and neurological toxicity (peripheral neuropathy, 93.8%). Events occurring in 50–80% of patients included thrombocytopenia (50.0%) and fatigue (50.0%). These toxicities are consistent with the known adverse event profile of the AG backbone regimen, and no unexpected toxicity types were observed following the addition of nimotuzumab.

Grade 3 events were limited to leukopenia (*n* = 2), nausea (*n* = 2), vomiting (*n* = 2), decreased appetite (*n* = 2), neutropenia (*n* = 1), and anemia (*n* = 1). Alopecia occurred in 81.2% of patients and was correctly graded as Grade 2 per NCI-CTCAE v5.0. These Grade 3 events resolved promptly following targeted interventions and did not lead to infectious complications, hemorrhagic events, or serious gastrointestinal injury. Notably, no hepatotoxicity was recorded (ALT and AST elevations: 0%), no constipation or diarrhea was observed, and no cases of bone pain were attributed to ≥Grade 2 severity. Importantly, peripheral neuropathy and anemia showed no evidence of cumulative toxicity; their incidence and severity remained stable across the median of 6 treatment cycles and did not lead to dose modifications or treatment discontinuation.

No serious adverse events (SAEs) were reported during the study period. The overall safety profile of the NTZ-AG regimen was consistent with that anticipated for AG-based therapy, and the addition of nimotuzumab did not appear to introduce novel or unexpected toxicities, supporting the clinical applicability of this combination in patients with advanced pancreatic cancer.

## Discussion

This single-arm, single-center exploratory Phase II trial represents one of the first prospective evaluations of the triple combination of nimotuzumab, nab-paclitaxel, and gemcitabine (NTZ-AG) as first-line therapy for advanced pancreatic cancer in an unselected patient population. In 16 patients with Stage IV PDAC who were not pre-screened for KRAS mutation status, the NTZ-AG regimen achieved an ORR of 68.75%, a DCR of 100%, a median PFS of 6.0 months, and a median OS of 12.0 months. The toxicity profile was predominantly Grade 1–2, with no Grade 4 or higher events and no treatment discontinuations, indicating acceptable tolerability.

**Figure 4:**
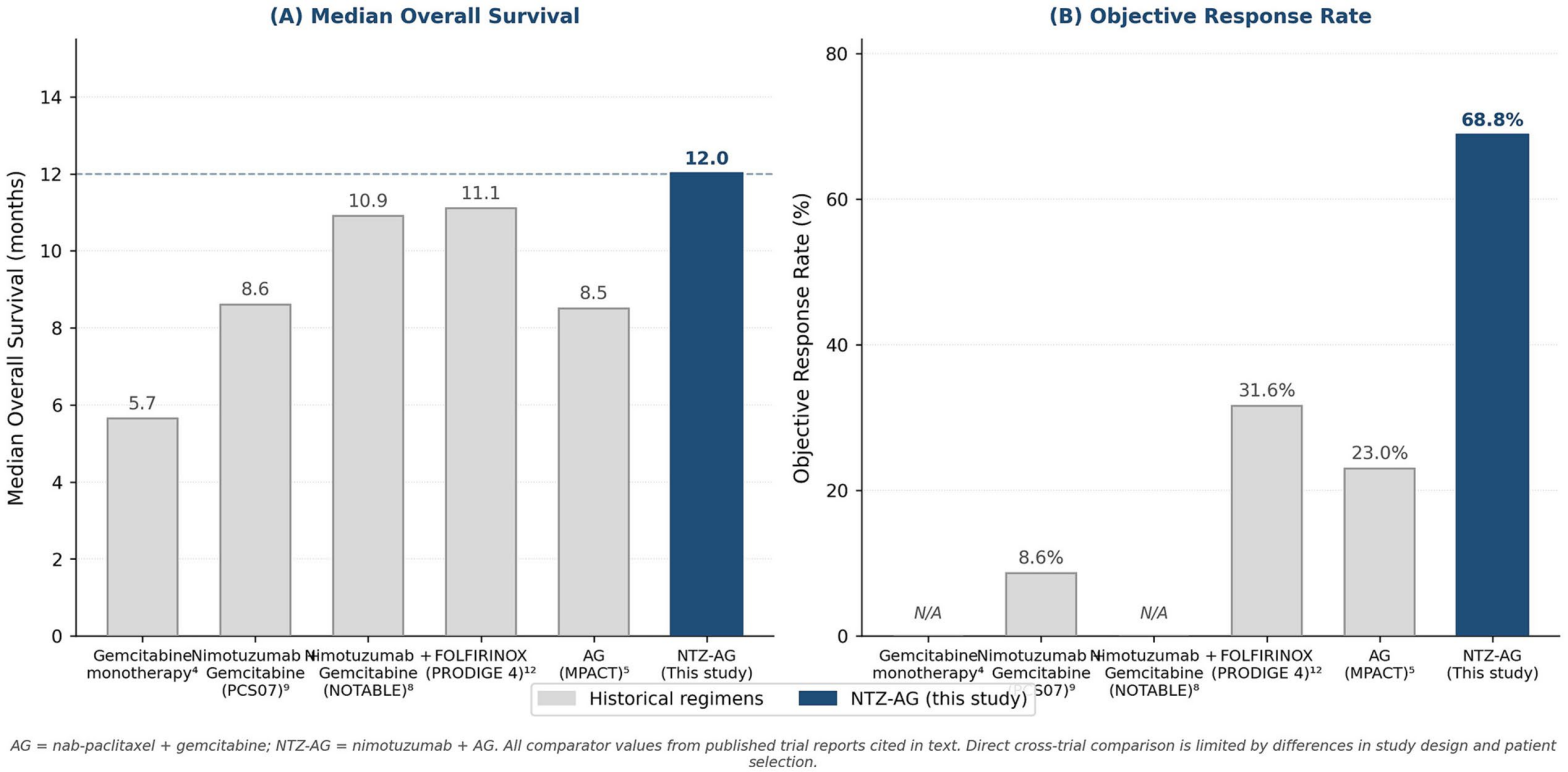
Comparison of median overall survival **(A)** and objective response rate **(B)** for the NTZ-AG regimen versus established first-line regimens for advanced pancreatic cancer. All comparator values are from published trial reports cited in the text. Direct cross-trial comparisons are limited by differences in patient selection and study design. AG = nab-paclitaxel + gemcitabine; NTZ-AG = nimotuzumab + AG; ORR = objective response rate; OS = overall survival.

The clinical activity observed with the NTZ-AG regimen compares favorably to historical benchmarks for first-line therapy in advanced pancreatic cancer (Figure 4). The ORR of 68.75% substantially exceeds the approximately 23% reported for AG in the MPACT trial (9) and the 8.6% reported for nimotuzumab plus gemcitabine in the PCS07 trial (14). The median OS of 12.0 months was also notably higher than observed in the MPACT trial (8.5 months) (9), the PCS07 trial (8.6 months) (14), and the NOTABLE trial (10.9 months) (15). While direct comparisons across trials are inherently limited by differences in patient selection, study design, and era of treatment, these data collectively suggest that the NTZ-AG regimen may represent a meaningful step forward in efficacy over available dual-agent regimens.

The mechanistic rationale for the NTZ-AG triple combination is multifaceted. The AG regimen offers a dual cytotoxic foundation: nab-paclitaxel, by binding to SPARC protein enriched in the tumor stroma, facilitates targeted drug accumulation within the tumor microenvironment while simultaneously disrupting the desmoplastic stromal barrier that restricts drug delivery; gemcitabine, in turn, exerts cytotoxic effects through inhibition of DNA synthesis and ribonucleotide reductase (20, 21). The addition of nimotuzumab introduces a targeted mechanism: as an anti-EGFR monoclonal antibody, nimotuzumab blocks EGFR-mediated downstream signaling (including the RAS/MAPK and PI3K/AKT/mTOR pathways), suppresses tumor cell proliferation, and induces apoptosis (10). Importantly, preclinical evidence suggests that nimotuzumab may augment the cytotoxic efficacy of gemcitabine by downregulating the expression of nucleotide excision repair proteins, thereby reducing the cell’s capacity to repair chemotherapy-induced DNA damage. The intermediate receptor affinity of nimotuzumab—a unique feature relative to cetuximab and panitumumab—may preferentially engage tumor cells with high EGFR density while sparing normal tissues, preserving therapeutic selectivity and minimizing dermatological toxicity (12).

An important practical advantage of the NTZ-AG regimen is its independence from molecular pre-screening. Unlike strategies contingent on KRAS wild-type status—as evaluated in the NOTABLE trial—the NTZ-AG combination can be initiated without awaiting genetic testing results, thereby avoiding delays that may be clinically significant in a disease as aggressive as PDAC. This characteristic is particularly relevant in healthcare settings where molecular diagnostics are not universally accessible, and is consistent with the observed DCR of 100% in the present cohort, suggesting broad anti-tumor activity regardless of mutational status. Although definitive conclusions about KRAS-independent activity cannot be drawn from the present data given the lack of prospective molecular profiling, the findings are hypothesis-generating for future biomarker-stratified studies.

The safety profile of the NTZ-AG regimen in the current study was broadly consistent with the established toxicity profile of the AG backbone. The predominant adverse events—anemia, neutropenia, peripheral neuropathy, nausea, and alopecia—are characteristic of nab-paclitaxel and gemcitabine-based chemotherapy and are anticipated and manageable in clinical practice. Notably, the addition of nimotuzumab did not appear to introduce any incremental toxicity burden beyond the AG regimen; in particular, the absence of significant dermatological toxicity (no skin rash or acneiform eruptions were recorded) is consistent with nimotuzumab’s intermediate EGFR affinity and its known favorable dermatological profile (12). The absence of Grade 4 toxicities and the lack of any treatment discontinuations further support the favorable tolerability of this regimen. These findings are in alignment with previous reports of nimotuzumab combined with gemcitabine in the PCS07 and NOTABLE trials (14, 15).

Several limitations of this study must be acknowledged. First, the sample size is small (*n* = 16), reflecting the challenges of recruitment in a single-center design, and the statistical power is insufficient to draw definitive conclusions. The failure to achieve the pre-specified sample of 31 patients further constrains the interpretation of results. Second, the single-arm design without a concurrent control group precludes direct causal attribution of observed outcomes to the study regimen; comparisons with historical controls are subject to selection bias and confounding. Third, prospective molecular profiling for KRAS, EGFR expression, and SPARC status was not systematically performed, limiting the capacity for biomarker-stratified subgroup analyses. Fourth, the relatively limited follow-up duration may underestimate the long-term survival benefit of the regimen, and maturity of survival data warrants additional follow-up. Finally, as a single-center study, generalizability to broader patient populations requires validation in multi-institutional settings.

As a single-arm, single-center Phase II study without a concurrent control arm, the present trial has inherent methodological limitations, including potential selection bias and the inability to isolate the contribution of nimotuzumab from the AG backbone with certainty. Cross-trial comparisons with historical data (e.g., ORR ~ 23% and median OS ~8.5 months with AG in the MPACT trial) are informative but must be interpreted cautiously due to differences in patient populations, era of treatment, and supportive care. Accordingly, the encouraging efficacy signals observed here (ORR 68.75%, DCR 100%, median OS 12.0 months) do not constitute definitive evidence of superiority. Rather, they provide preliminary evidence of meaningful anti-tumor activity and a favorable safety profile in an unselected population, generating a strong rationale for further investigation in larger randomized studies.

These limitations notwithstanding, the present study provides clinically meaningful preliminary evidence that the NTZ-AG regimen exhibits substantial anti-tumor activity in unselected patients with advanced PDAC.

Additional limitations include the small sample size (*n* = 16, 51.6% of the planned 31), the lack of prospective molecular profiling, and the absence of formal quality-of-life assessments. Archival tumor tissue sufficient for retrospective KRAS or EGFR testing was available in only three patients (18.75%), precluding any meaningful biomarker subgroup analysis. Prospective molecular profiling for KRAS, EGFR expression, or SPARC status was not performed, as the study was deliberately designed to evaluate the NTZ-AG regimen in an unselected population to reflect real-world clinical practice. Formal quality-of-life assessments using validated instruments (e.g., EORTC QLQ-C30) were not performed. Given the intensity of the triple-drug regimen, this represents a limitation; based on the observed favorable safety profile—predominantly Grade 1–2 toxicities that were non-cumulative and stable over the median of 6 cycles, with no Grade 4 events or treatment discontinuations, global health status and functional scales were likely reasonably maintained overall, particularly among patients who achieved disease control.

Future investigative directions should include a multicenter randomized controlled Phase III trial comparing NTZ-AG versus AG in unselected patients; prospective biomarker stratification studies to identify the patients most likely to benefit (based on KRAS, EGFR, and SPARC expression); evaluation of the NTZ-AG regimen in the neoadjuvant setting for borderline-resectable pancreatic cancer; and translational research to elucidate the mechanisms by which nimotuzumab may circumvent resistance associated with KRAS mutations. Prospective QoL evaluation should also be incorporated in future studies, including the planned Phase III trial, which we intend to conduct and will report separately.

## Conclusion

In this preliminary Phase II study, the NTZ-AG regimen, comprising nimotuzumab combined with nab-paclitaxel and gemcitabine demonstrated encouraging anti-tumor activity and a manageable safety profile as first-line therapy for advanced pancreatic cancer in an unselected patient population. While the NTZ-AG regimen demonstrated encouraging anti-tumor activity and an acceptable safety profile in this exploratory Phase II setting, definitive confirmation of its clinical benefit will require a multicenter randomized controlled Phase III trial comparing NTZ-AG versus AG, which we plan to conduct and will report separately in due course. Taken together, the results of the present study establish a scientific rationale and provide supportive data for the conduct of larger, prospective, randomized controlled trials with biomarker stratification, which will be necessary to definitively establish the clinical value of the NTZ-AG regimen and to optimize patient selection for this promising therapeutic approach.

## Data Availability

The original contributions presented in the study are included in the article/Supplementary material, further inquiries can be directed to the corresponding authors.

## Author’s note

We sincerely thanks Professor Shaozhong He for provinding subjects for this study.
