# Supplementary material for: Nimotuzumab combined with gemcitabine and nab-paclitaxel as first-line therapy for advanced pancreatic cancer: a single-arm, single-center Phase II prospective study
Source: Front Med (Lausanne). 2026 May 29;13:1838482. doi: 10.3389/fmed.2026.1838482 (PMC13261391; doi:10.3389/fmed.2026.1838482)
Supplement: Supplementary file 1 [file Supplementary_file_1.docx]

**Supplemental Table S1.** Exploratory subgroup analysis of objective response rate (ORR) and overall survival (OS) by age and sex (N=16)

| **Subgroup** | **N** | **ORR, n (%)** | **Median OS, months (95% CI)** |
| --- | --- | --- | --- |
| Age |  |  |  |
| <65 years | 9 | 7 (77.8) | 13.0 (8.5–17.5) |
| ≥65 years | 7 | 4 (57.1) | 10.5 (7.0–14.0) |
| Sex |  |  |  |
| Male | 10 | 7 (70.0) | 12.5 (8.0–17.0) |
| Female | 6 | 4 (66.7) | 11.0 (6.5–15.5) |
| Overall | 16 | 11 (68.75) | 12.0 (8.35–15.66) |

**Note:** Analyses are exploratory and descriptive only. All ORR based on confirmed responses per RECIST v1.1.
